# Supplementary material for: Anaerobic Carbon Monoxide Uptake by Microbial Communities in Volcanic Deposits at Different Stages of Successional Development on O-yama Volcano, Miyake-jima, Japan
Source: Microorganisms. 2020 Dec 22;9(1):12. doi: 10.3390/microorganisms9010012 (PMC7822213; doi:10.3390/microorganisms9010012)
Supplement: Supplementary file 1 [file microorganisms-09-00012-s001.zip › Miyake_supplementary_tables.docx]

Table S1. Phyla present at mean relative abundances > 1% for No CO 25°C at sites CL, IG-7 and OY.

| Phylum | No CO 25°C | | | | | | | | |
| --- | --- | --- | --- | --- | --- | --- | --- | --- | --- |
|  | CL 1 | CL 2 | CL 3 | IG-7 1 | IG-7 2 | IG-7 3 | OY 1 | OY 2 | OY 3 |
| Proteobacteria | 39.7 | 39.9 | 41.9 | 0.78 | 2.15 | 0.43 | 34.3 | 33.3 | 36.2 |
| Acidobacteria | 22.8 | 21.7 | 21.8 | 39.8 | 43.6 | 39.9 | 15.1 | 13.9 | 17.1 |
| Verrumicrobia | 12.9 | 13.5 | 12.6 | 1.89 | 1.81 | 2.22 | 2.03 | 1.68 | 1.69 |
| Bacteroidetes | 8.94 | 8.19 | 7.52 | 5.82 | 7.24 | 7.78 | 4.31 | 2.73 | 1.59 |
| Chloroflexi |  |  |  | 8.81 | 6.46 | 7.50 | 21.5 | 23.7 | 18.6 |
| Actinobacteria | 1.52 | 1.65 | 2.12 | 10.4 | 7.24 | 7.78 | 4.58 | 4.54 | 6.04 |
| Planctomyces | 6.85 | 6.94 | 7.00 | 4.31 | 4.45 | 2.16 | 7.60 | 7.31 | 7.08 |
| Candidate Phyla WPS-2 |  |  |  | 6.42 | 6.26 | 6.22 | 2.67 | 3.44 | 2.38 |
| Patescibacteria | 1.68 | 1.73 | 1.56 |  |  |  | 1.57 | 1.62 | 1.87 |
| Thaumarchaeota |  |  |  |  |  |  | 2.17 | 2.67 | 2.85 |
| Planctomyces | 5.98 | 5.05 | 4.75 |  |  |  |  |  |  |
| Rokubacteria | 1.87 | 2.12 | 1.61 |  |  |  |  |  |  |
| Latescibacteria | 1.29 | 1.31 | 2.32 |  |  |  |  |  |  |

Table S2. Genera present at mean relative abundances > 1% for No CO 25˚C.

| Genus | No CO 25°C | | | | | | | | |
| --- | --- | --- | --- | --- | --- | --- | --- | --- | --- |
|  | CL 1 | CL 2 | CL 3 | IG-7 1 | IG-7 2 | IG-7 3 | OY 1 | OY 2 | OY 3 |
| *Pseudolabrys* | 6.77 | 7.51 | 6.99 |  | 7.51 | 6.99 |  |  |  |
| *Bradyrhizobium* | 4.88 | 5.01 | 6.26 |  | 5.01 | 6.26 | 3.79 | 2.46 | 2.16 |
| *Candidatus Udaeobacter* | 12.9 | 14.1 | 15.1 |  | 14.1 | 15.1 |  |  |  |
| *Chthoniobacter* | 2.20 | 1.90 | 1.81 |  | 1.90 | 1.81 |  |  |  |
| *Candidatus Xiphinematobacter* | 6.21 | 6.80 | 5.92 |  | 6.80 | 5.92 |  |  |  |
| *Acidibacter* | 3.39 | 3.54 | 3.81 | 5.15 | 4.56 | 5.33 | 4.86 | 4.46 | 2.17 |
| Acidobacteria *JGI 0001001-H03* | 5.49 | 4.58 | 4.50 |  | 4.58 | 4.50 |  |  |  |
| *Flavobacterium* | 2.63 | 2.71 | 2.19 |  | 2.71 | 2.19 |  |  |  |
| *ADurb.Bin063-1* | 2.28 | 2.41 | 1.93 |  | 2.41 | 1.93 |  |  |  |
| *Reyranella* | 2.21 | 1.80 | 2.17 |  | 1.80 | 2.17 |  |  |  |
| *Haliangium* | 2.12 | 2.50 | 1.93 | 2.78 | 0 | 2.75 |  |  |  |
| Acidobacteria *RB41* | 3.36 | 3.00 | 2.50 |  | 3.00 | 2.50 |  |  |  |
| *Xylophilus* | 1.87 | 1.53 | 1.28 |  | 1.53 | 1.28 |  |  |  |
| *Candidatus Solibacter* | 2.05 | 1.78 | 1.62 | 4.96 | 2.74 | 4.19 |  |  |  |
| *Bryobacter* | 2.06 | 2.01 | 1.45 |  | 2.01 | 1.45 |  |  |  |
| *Acidothermus* |  |  |  | 3.30 | 2.78 | 1.68 |  |  |  |
| *Roseiarcus* |  |  |  | 2.11 | 4.20 | 3.87 |  |  |  |
| *Bryobacter* |  |  |  | 5.71 | 4.93 | 8.73 | 0 | 3.01 | 4.52 |
| *Burkholderia-Caballeronia-Paraburkholderia* |  |  |  | 7.20 | 4.76 | 5.92 |  |  |  |
| *Pajaroellobacter* |  |  |  | 2.10 | 6.42 | 3.74 |  |  |  |
| *Occallatibacter* |  |  |  | 4.09 | 0 | 5.91 |  |  |  |
| *Mucilaginibacter* |  |  |  | 4.81 | 3.21 | 3.24 |  |  |  |
| *Terracidiphilus* |  |  |  | 4.02 | 3.60 | 2.90 |  |  |  |
| *Sphingomonas* |  |  |  | 2.76 | 2.16 | 0 |  |  |  |
| *Opitutus* |  |  |  |  |  |  | 4.33 | 1.38 | 2.31 |
| Ktedonobacteraceae *1921-2* |  |  |  |  |  |  | 2.24 | 2.95 | 1.57 |
| *Noviherbasspirillum* |  |  |  |  |  |  | 0 | 2.28 | 2.96 |
| *Candidatus Nitrosotalea* |  |  |  |  |  |  | 4.69 | 4.81 | 5.26 |
| *Phenylobacterium* |  |  |  |  |  |  | 5.27 | 2.21 | 7.72 |
| *Alkanibacter* |  |  |  |  |  |  | 1.54 | 6.04 | 1.23 |
| *Nevskia* |  |  |  |  |  |  | 4.98 | 5.59 | 4.67 |
| *Limnobacter* |  |  |  |  |  |  | 0 | 5.38 | 1.26 |
| *Pseudomonas* |  |  |  |  |  |  | 0 | 0 | 5.12 |
| *Aquabacterium* |  |  |  |  |  |  | 4.83 | 3.05 | 1.35 |
| *Marmoricola* |  |  |  |  |  |  | 1.47 | 2.68 | 4.58 |
| *Anaeromyxobacter* |  |  |  |  |  |  | 3.50 | 2.67 | 0 |
| *Turneriella* |  |  |  |  |  |  | 0 | 3.22 | 1.34 |
| *Ralstonia* |  |  |  |  |  |  | 10.6 | 2.35 | 0 |

Table S3. Phyla present at mean relative abundances > 1% for 25% CO 25°C at sites CL, IG-7 and OY. * indicates replicates that had no CO uptake activity for the community change assay.

| Phylum | 25% CO 25°C | | | | | | | | |
| --- | --- | --- | --- | --- | --- | --- | --- | --- | --- |
|  | CL 1 | CL 2 | CL 3 | IG-7 1 | IG-7 2 | IG-7 3 | OY 1 | OY 2 | OY 3 |
| Firmicutes | 2.57 | 1.04 | 1.60 | 25.5 | 46.4 | 27.3 |  |  |  |
| Proteobacteria | 41.9 | 41.6 | 41.6 | 32.2 | 22.8 | 27.1 | 23.2 | 34.5 | 40.6 |
| Acidobacteria | 22.2 | 22.3 | 24.0 | 14.4 | 10.4 | 11.8 | 8.89 | 10.7 | 11.2 |
| Verrumicrobia | 11.9 | 10.9 | 12.2 | 3.51 | 2.02 | 3.80 | 11.0 | 3.88 | 3.60 |
| Bacteroidetes | 5.69 | 8.16 | 6.23 | 4.99 | 2.00 | 3.36 | 23.1 | 10.7 | 10.8 |
| Chloroflexi | 2.04 | 1.62 | 1.18 | 8.40 | 5.48 | 12.5 | 21.8 | 26.0 | 22.9 |
| Actinobacteria | 2.13 | 2.08 | 1.56 | 3.99 | 3.80 | 5.55 | 1.97 | 3.53 | 1.73 |
| Planctomyces | 6.41 | 6.39 | 5.97 | 4.60 | 4.72 | 6.09 | 4.53 | 5.17 | 4.15 |
| Candidate Phyla WPS-2 |  |  |  |  |  |  | 1.90 | 4.35 | 3.06 |
| Patescibacteria |  |  |  |  |  |  | 0.47 | 1.20 | 2.43 |
| Thaumarchaeota |  |  |  |  |  |  | 1.41 | 1.30 | 2.08 |
| Rokubacteria | 2.01 | 1.89 | 1.83 |  |  |  |  |  |  |
| Latescibacteria | 12.0 | 1.42 | 1.72 |  |  |  |  |  |  |

Table S4. Genera present at mean relative abundances > 1% for 25% CO 25°C at sites CL,

IG-7 and OY, * indicates replicates that had no CO uptake activity for the community change assay

| Genus | 25% CO 25°C | | | | | | | | |
| --- | --- | --- | --- | --- | --- | --- | --- | --- | --- |
|  | CL 1 | CL 2 | CL 3 | IG-7 1 * | IG-7 2 * | IG-7 3 * | OY 1 * | OY 2 * | OY 3 * |
| *Pseudolabrys* | 6.69 | 7.33 | 6.82 |  |  |  | 0 | 5.13 | 2.13 |
| *Bradyrhizobium* | 6.54 | 6.54 | 5.79 |  |  |  | 0 | 13.5 | 4.47 |
| *Candidatus Udaeobacter* | 16.4 | 14.4 | 15.8 |  |  |  |  |  |  |
| *Acidibacter* | 3.76 | 3.47 | 4.41 |  |  |  |  |  |  |
| Acidobacteria *JGI 0001001-H03* | 2.65 | 3.87 | 4.35 |  |  |  |  |  |  |
| *Pelosinus* | 4.27 | 0 | 2.17 |  |  |  |  |  |  |
| *Flavobacterium* | 0 | 3.17 | 2.11 |  |  |  |  |  |  |
| Verrucomicrobia *ADurb.Bin063-1* | 2.94 | 1.89 | 2.09 |  |  |  |  |  |  |
| *Reyranella* | 2.32 | 2.64 | 2.55 |  |  |  |  |  |  |
| *Haliangium* | 1.47 | 2.41 | 1.98 |  |  |  |  |  |  |
| Acidobacteria *RB41* | 2.40 | 2.36 | 2.22 |  |  |  |  |  |  |
| *Aquisphaera* | 2.28 | 1.54 | 1.25 |  |  |  |  |  |  |
| *Xylophilus* | 1.60 | 1.70 | 1.37 |  |  |  |  |  |  |
| *Candidatus Solibacter* | 1.60 | 1.78 | 1.52 |  |  |  | 1.32 | 3.32 | 2.87 |
| *Desulfitobacterium* |  |  |  | 42.0 | 12.4 | 41.3 |  |  |  |
| *Clostridium sensu stricto 12* |  |  |  | 0 | 23.5 | 0 |  |  |  |
| *Clostridium sensu stricto 10* |  |  |  | 0 | 12.4 | 3.03 |  |  |  |
| *Candidatus Koribacter* |  |  |  | 7.54 | 3.18 | 2.76 |  |  |  |
| *Acidothermus* |  |  |  | 0 | 2.78 | 4.93 |  |  |  |
| *Fonticella* |  |  |  | 0 | 4.28 | 0 |  |  |  |
| *Sediminibacterium* |  |  |  | 0 | 3.60 | 3.13 | 42.1 | 2.13 | 32.2 |
| *Roseiarcus* |  |  |  | 0 | 2.02 | 2.93 |  |  |  |
| *Opitutus* |  |  |  |  |  |  | 16.7 | 6.79 | 5.28 |
| Ktedonobacteraceae *1921-2* |  |  |  |  |  |  | 1.78 | 5.15 | 2.35 |
| *Bryobacter* |  |  |  |  |  |  | 2.66 | 4.73 | 0 |
| *Halophaga* |  |  |  |  |  |  | 1.83 | 2.40 | 3.49 |
| *Noviherbasspirillum* |  |  |  |  |  |  | 0 | 4.66 | 1.95 |
| *Ralstonia* |  |  |  |  |  |  | 1.73 | 2.96 | 1.01 |

Table S5. Phyla present at mean relative abundances > 1% for No CO 60°C at sites CL, IG-7 and OY.

| Phylum | No CO 60°C | | | | | | | | |
| --- | --- | --- | --- | --- | --- | --- | --- | --- | --- |
|  | CL 1 | CL 2 | CL 3 | IG-7 1 | IG-7 2 | IG-7 3 | OY 1 | OY 2 | OY 3 |
| Firmicutes | 35.9 | 60.6 | 46.3 | 98.2 | 99.2 | 96.3 | 98.3 | NA | 91.7 |
| Proteobacteria | 38.5 | 21.3 | 31.7 |  |  |  | 0.21 | NA | 6.22 |
| Acidobacteria | 5.41 | 4.60 | 4.85 |  |  |  |  |  |  |
| Verrumicrobia | 3.85 | 2.09 | 3.09 |  |  |  |  |  |  |
| Bacteroidetes | 2.40 | 0.43 | 1.98 |  |  |  |  |  |  |
| Chloroflexi | 2.63 | 3.67 | 2.42 | 13.5 | 0.85 | 3.57 |  |  |  |
| Actinobacteria | 4.89 | 2.41 | 3.32 |  |  |  | 0 | NA | 2.07 |
| Rokubacteria | 1.99 | 1.43 | 1.98 |  |  |  |  |  |  |

Table S6. Genera present at mean relative abundances > 1% for No CO 60°C at sites CL, IG-7 and OY.

| Genus | No CO 60°C | | | | | | | | |
| --- | --- | --- | --- | --- | --- | --- | --- | --- | --- |
|  | CL 1 | CL 2 | CL 3 | IG-7 1 | IG-7 2 | IG-7 3 | OY 1 | OY 2 | OY 3 |
| *Geobacillus* | 4.12 | 4.09 | 5.25 |  |  |  |  |  |  |
| *Paenibacillus* | 3.28 | 23.4 | 6.67 |  |  |  |  |  |  |
| *Tumebacillus* | 9.53 | 18.9 | 6.25 | 17.8 | 0 | 9.87 | 0 | NA | 9.04 |
| *Bacillus* | 4.19 | 9.88 | 6.95 | 2.04 | 3.68 | 5.06 |  |  |  |
| *Pseudolabrys* | 4.61 | 3.18 | 6.93 |  |  |  |  |  |  |
| *Bradyrhizobium* | 3.80 | 3.72 | 5.62 |  |  |  |  |  |  |
| *Candidatus Udaeobacter* | 4.83 | 4.22 | 5.76 |  |  |  |  |  |  |
| *Anoxybacillus* | 1.74 | 0 | 5.65 |  |  |  |  |  |  |
| *Caldanaerobius* | 0 | 0 | 3.75 |  |  |  |  |  |  |
| *Thermoanaerobacterium* | 18.7 | 0 | 0 | 0 | 86.8 | 41.6 | 15.6 | NA | 0 |
| *Symbiobacterium* | 7.01 | 0 | 0 |  |  |  |  |  |  |
| *Effusibacillus* |  |  |  | 2.54 | 1.05 | 2.76 |  |  |  |
| *Alicyclobacillus* |  |  |  | 74.6 | 36.2 | 0 |  |  |  |
| *Kyrpidia* |  |  |  |  |  |  | 65.7 | NA | 53.7 |
| *Brevibacillus* |  |  |  |  |  |  | 1.73 | NA | 7.97 |

| Phylum | 25% CO 60°C | | | | | | | | |
| --- | --- | --- | --- | --- | --- | --- | --- | --- | --- |
|  | CL 1 | CL 2 | CL 3 | IG-7 1 * | IG-7 2 * | IG-7 3 | OY 1 | OY 2 * | OY 3* |
| Firmicutes | 60.8 | 50.8 | 46.8 | 95.2 | 98.9 | 98.0 | 100 | 99.1 | 100 |
| Proteobacteria | 25.1 | 28.7 | 30.5 | 2.91 | 0.61 | 1.19 |  |  |  |
| Acidobacteria | 3.21 | 3.84 | 3.98 |  |  |  |  |  |  |
| Verrumicrobia | 1.53 | 2.13 | 2.69 |  |  |  |  |  |  |
| Chloroflexi | 2.42 | 3.50 | 4.06 |  |  |  |  |  |  |
| Actinobacteria | 2.51 | 3.09 | 4.40 |  |  |  |  |  |  |
| Rokubacteria | 0 | 2.57 | 1.89 |  |  |  |  |  |  |

Table S7. Phyla present at mean relative abundances > 1% for 25% CO 60°C at sites CL, IG-7 and OY. * indicates replicates that had no CO uptake activity for the community change assay.

Table S8. Genera present at mean relative abundances > 1% for 25% CO 60°C at sites CL, IG-7 and OY. * indicates replicates that had no CO uptake activity for the community change assay.

| Genus | 25% CO 60°C | | | | | | | | |
| --- | --- | --- | --- | --- | --- | --- | --- | --- | --- |
|  | CL 1 | CL 2 | CL 3 | IG-7 1 * | IG-7 2 * | IG-7 3 | OY 1 | OY 2 * | OY 3 * |
| *Geobacillus* | 27.2 | 22.1 | 21.0 |  |  |  |  |  |  |
| *Paenibacillus* | 12.0 | 17.3 | 12.2 |  |  |  |  |  |  |
| *Tumebacillus* | 10.7 | 6.12 | 5.72 | 0 | 0 | 66.3 | 13.8 | 0 | 0 |
| *Bacillus* | 8.04 | 10.1 | 8.76 | 33.6 | 9.41 | 1.32 |  |  |  |
| *Tuberibacillus* | 5.11 | 1.84 | 8.59 |  |  |  |  |  |  |
| *Pseudolabrys* | 3.37 | 4.91 | 5.03 |  |  |  |  |  |  |
| Ktedonobacteraceae *1959-1* | 1.59 | 2.84 | 3.70 |  |  |  |  |  |  |
| *Bradyrhizobium* | 2.17 | 3.02 | 3.45 |  |  |  |  |  |  |
| *Candidatus Udaeobacter* | 1.68 | 2.73 | 3.28 |  |  |  |  |  |  |
| *Anoxybacillus* | 0 | 2.50 | 1.37 |  |  |  |  |  |  |
| *Caldanaerobius* | 8.44 | 0 | 0 |  |  |  |  |  |  |
| *Pedomicrobium* | 1.47 | 2.25 | 1.64 |  |  |  |  |  |  |
| *Cohnella* | 1.00 | 2.15 | 0 |  |  |  |  |  |  |
| *Thermoanaerobacterium* |  |  |  | 0 | 42.8 | 0 |  |  |  |
| *Effusibacillus* |  |  |  | 6.07 | 27.3 | 0 |  |  |  |
| *Alicyclobacillus* |  |  |  | 18.8 | 5.22 | 0 |  |  |  |
| *Moorella* |  |  |  |  |  |  | 35.1 | 25.1 | 19.3 |

Table S9. Alpha diversity metrics for all treatments (T0, No CO 25°C, 25% CO 25°C, No CO 60°C and 25% CO 60°C) for Sites CL, IG-7 and OY. * indicates replicates that had no CO uptake activity for the community change assay. NA values were samples that were removed after rarefying to the minimum sample depth (10,609).

| Treatment | Chao1 | | | Shannon | | |
| --- | --- | --- | --- | --- | --- | --- |
| T0 |  |  |  |  |  |  |
| CL | 1804.5 | 1566.9 | 1422.1 | 6.66 | 6.57 | 6.47 |
| IG-7 | 805.8 | 1162.5 | 1172.6 | 6.00 | 6.39 | 6.33 |
| OY | 533.4 | 504.0 | 497.2 | 4.96 | 4.87 | 5.05 |
| No CO 25°C |  |  |  |  |  |  |
| CL | 1659.6 | 1772.3 | 1753.5 | 6.72 | 6.70 | 6.66 |
| IG-7 | 1252.7 | 1304.2 | 1199.6 | 6.39 | 6.52 | 6.40 |
| OY | 476.8 | 652.6 | 506.9 | 4.99 | 5.01 | 5.06 |
| 25% CO 25°C |  |  |  |  |  |  |
| CL | 1420.3 | 1885.1 | 1712.6 | 6.39 | 6.70 | 6.59 |
| IG-7 | 1044.4* | 1014.8* | 1156.9* | 5.10* | 5.00* | 5.52* |
| OY | 476.1* | 415.5* | 428.4* | 4.09* | 4.40* | 4.42* |
| No CO 60°C |  |  |  |  |  |  |
| CL | 1819.2 | 1137.6 | 1584.2 | 5.87 | 4.54 | 5.04 |
| IG-7 | 90.8 | 82 | 74.2 | 1.89 | 0.91 | 2.01 |
| OY | NA | NA | NA | NA | NA | NA |
| 25% CO 60°C |  |  |  |  |  |  |
| CL | 1150.1 | 1135.9 | 1467.9 | 4.82 | 5.38 | 5.46 |
| IG-7 | NA* | 49* | 116.2 | NA* | 2.22* | 1.18 |
| OY | NA | NA | NA | NA | NA | NA |
